# Complementary ACSL isoforms contribute to a non-Warburg advantageous energetic status characterizing invasive colon cancer cells

**DOI:** 10.1038/s41598-017-11612-3

**Published:** 2017-09-11

**Authors:** Ruth Sánchez-Martínez, Silvia Cruz-Gil, María Soledad García-Álvarez, Guillermo Reglero, Ana Ramírez de Molina

**Affiliations:** Molecular Oncology and Nutritional Genomics of Cancer Group, IMDEA Food Institute, CEI UAM+CSIC, E28049 Madrid, Spain

## Abstract

Metabolic reprogramming is one of cancer hallmarks. Here, we focus on functional differences and individual contribution of acyl coA synthetases (ACSL) isoforms to the previously described ACSL/stearoyl-CoA desaturase (ACSL1/ACSL4/SCD) metabolic network causing invasion and poor prognosis in colorectal cancer (CRC). ACSL4 fuels proliferation and migration accompanied by a more glycolytic phenotype. Conversely, ACSL1 stimulates invasion displaying a lower basal respiratory rate. Acylcarnitines elevation, polyunsaturated fatty acids (PUFA) lower levels, and monounsaturated fatty acids (MUFA) upregulation characterize the individual overexpression of ACSL1, ACSL4 and SCD, respectively. However, the three enzymes simultaneous overexpression results in upregulated phospholipids and urea cycle derived metabolites. Thus, the metabolic effects caused by the network are far from being caused by the individual contributions of each enzyme. Furthermore, ACSL/SCD network produces more energetically efficient cells with lower basal respiration levels and upregulated creatine pathway. These features characterize other invasive CRC cells, thus, ACSL/SCD network exemplifies specific metabolic adaptations for invasive cancer cells.

## Introduction

Cancer energy relies on metabolic editing to fuel malignant transformation^1^. A great deal of effort has been done to characterize tumours metabolic phenotypes and new oncometabolites are constantly being described as markers of the disease^2^. Besides well-known carbohydrate metabolism alterations, it is becoming clear that there is an increasing range of metabolic adaptations that tumours can use to sustain their growth^3–9^.

Metabolic changes in cancer cells are often linked to growth and survival pathways driving different aspects of tumorigenesis. For instance, glycolytic behaviour associates with Akt and Erk pathways^10–13^, while *Myc* oncogene could govern glutamine addiction^14^. Alterations in lipid metabolism, both catabolic and anabolic, are part of the metabolic reprogramming that occurs in tumour cells in response to gene mutations, loss of tumour suppressors and epigenetic modifications^15,16^. Fatty acid (FA) metabolism enzymes have been found to be essential for neoplastic growth^17–20^ as well as lipid signalling triggers key tumorigenic pathways^21–23^.

Interconnection of metabolic pathways allows that metabolic enzymes deregulation in cancer exert unexpected effects on non-directly related routes^24^. Besides, cross-talk with tumorigenic pathways can cause activation of further metabolic routes triggered by core cancer signalling. This way, metabolic enzymes deregulation not only affect the proportion of their expected substrates and products as well as their immediate pathways. In some cases, substantial changes in unexpected parallel metabolic routes can be observed, allowing the connection with cell cycle regulation, redox management and other changes favouring different tumour cells characteristics^25,26^.

We have previously described a lipid network able to trigger epithelial-mesenchymal transition (EMT) and invasion, which is overexpressed in colorectal cancer (CRC) patients with poorer outcomes^19^. This network comprises ACSL1 and ACSL4, members of the fatty acid activating enzymes acyl coA synthetases (ACSL), critical for lipid synthesis, modification and β-oxidation^27^; and the stearoyl-CoA desaturase (SCD), the main enzyme controlling the rate of saturated (SFA) vs unsaturated fatty acids (MUFA)^28^, crucial for cancer cells^29^. These enzymes have been related to the prognosis and progression of several malignancies^30–36^. Despite ACSL isoforms can catalyse the same reaction, to bind a molecule of Acetyl–CoA to a fatty acid giving rise to an Acyl-CoA, there is increasing evidence for a specialization in the substrates, functions and cellular localizations. ACSL1 has been reported to be more inclined to triglyceride synthesis^37,38^. In contrast, ACSL4, that prefers longer polyunsaturated fatty acids (PUFA) as substrates such as arachidonic acid, has been proposed to channel FA towards phospholipids^39^. Here we further analyse the individual contributions of each enzyme to the ACSL/SCD network and the metabolic characteristics accompanying ACSL/SCD invasive cells. We present an example on how deregulation of metabolic enzymes gives rise to global metabolic changes that derive into specific ways of tumour fuelling associated with the invasive features of cancer cells.

## Results

### Metabolic differences correspond to diverse protumorigenic features conferred by ACSL1 and ACSL4 isoforms

**Figure 1:**
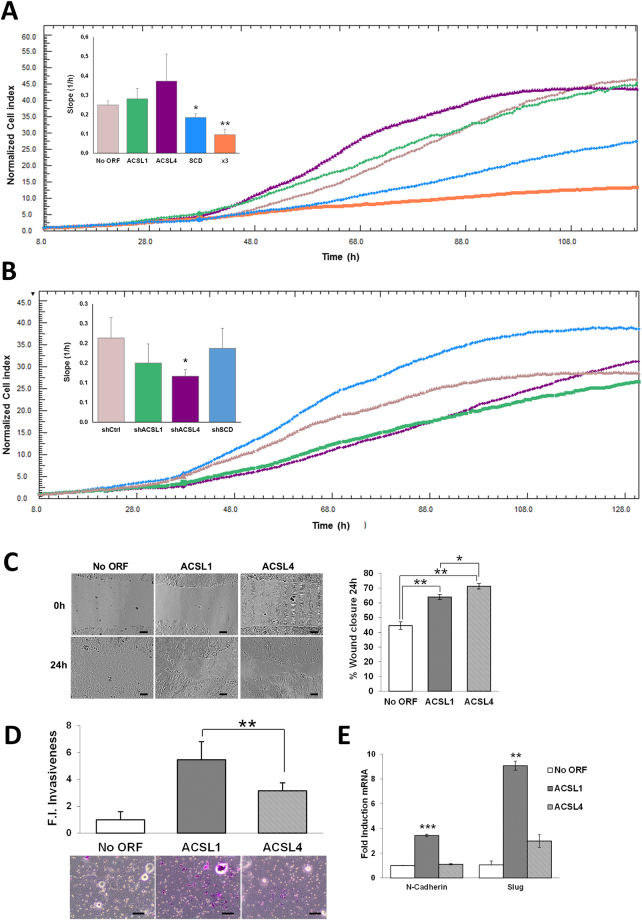
Comparison of protumorigenic capabilities conferred by ACSL isoforms to CRC cells. (**A**) Real-time monitoring of cell proliferation for cells overexpressing ACSL1, ACSL4, SCD, both individually or simultaneously (x3). (**B**) Real-time monitoring of cell proliferation for cells stably expressing shRNAs for ACSL1 (shACSL1), ACSL4 (shACSL4), SCD (shSCD) or scramble (shCtrl). Representative experiments are shown. Bar chart indicates the curves slope (proliferation rate) as the average of 3 independent experiments (n = 3). (**C**) Phase contrast pictures of wound healing assay comparing migratory capacities of No ORF, ACSL1 and ACSL4 cells. Quantification (right panel) shows the different behaviour of control, ACSL1 and ACSL4 cells at 24 hours of wound closure. Scale bars, 100 μm. (**D**) Boyden chamber transwell assay of 48 hours of invasion through Matrigel. Migratory cells were quantified as the average number of cells found in five random microscope fields in three independent inserts. Scale bars, 50 μm. (**E**) RT-QPCR analysis of mesenchymal genes (*N-Cadherin* and *Slug*) for ACSL1 and ACSL4 cells compared to levels in No ORF control cells. Experiments were performed in triplicates (n = 3). Results represent the mean ± SD (n = 3). *p < 0.05, **p < 0.01, ***p < 0.001.

In an earlier report, we described an ACSL1/ACSL4/SCD network causing EMT and invasion in CRC cells^19^. To address more in detail the individual contributions of each enzyme integrating the ACSL/SCD axis we started investigating the differences among ACSL1 and ACSL4 isoforms. First, using DLD-1 CRC cells stably overexpressing ACSL1 or ACSL4 proteins (ACSL1 or ACSL4 cells)^19^ we assayed cell proliferation. We used XCelligence technology to monitor real-time cell proliferation of these cell lines. ACSL4 overexpression caused the highest increase in proliferation when compared to control No ORF cells (Fig. 1A). Accordingly, the use of shRNAs against ACSLs (Supplementary Figure 1) caused the opposite effect, being again ACSL4 the isoform whose depletion caused the strongest effect on proliferation (Fig. 1B). The same tendency was observed in ACSL1 overexpressing or depleted cells, however, the effect was less marked. Specially, ACSL1 cells almost proliferated at a similar rate to the No ORF control cells. Curiously, SCD caused a reverse effect, decreasing proliferation rate upon overexpression (SCD cells^19^) and a proliferation increase in the case of shSCD cells (Fig. 1A,B and Supplementary Figure 1). Wound healing assays are normally performed to assay migratory capacity. However, wounds can be closed by unidirectional migration of cohesive epithelial sheets that can be related to proliferation abilities. Would healing assays show that ACSL4 cells were able to close the wound faster than the ones overexpressing ACSL1 (Fig. 1C), displaying this collective unidirectional migration of cohesive epithelial sheets that could be related to the stimulatory effect of ACSL4 on proliferation (Fig. 1A). Conversely, significantly more ACSL1 cells were able to invade through matrigel when compared to ACSL4 or poorly invasive DLD-1 No ORF control cells (Fig. 1D). Correspondingly, ACSL1 overexpression but not ACSL4 stimulated *N-cadherin* or *Slug* expression (Fig. 1E), well-known EMT markers involved in cancer invasion and metastasis^40,41^. This mesenchymal tendency of ACSL1 cells could lead them to close the wound more efficiently than No ORF control cells as can be inferred from the wound closure morphology (Fig. 1C). Taken together, these results highlight the different contributions to cancer cell features of both isoforms.

**Figure 2:**
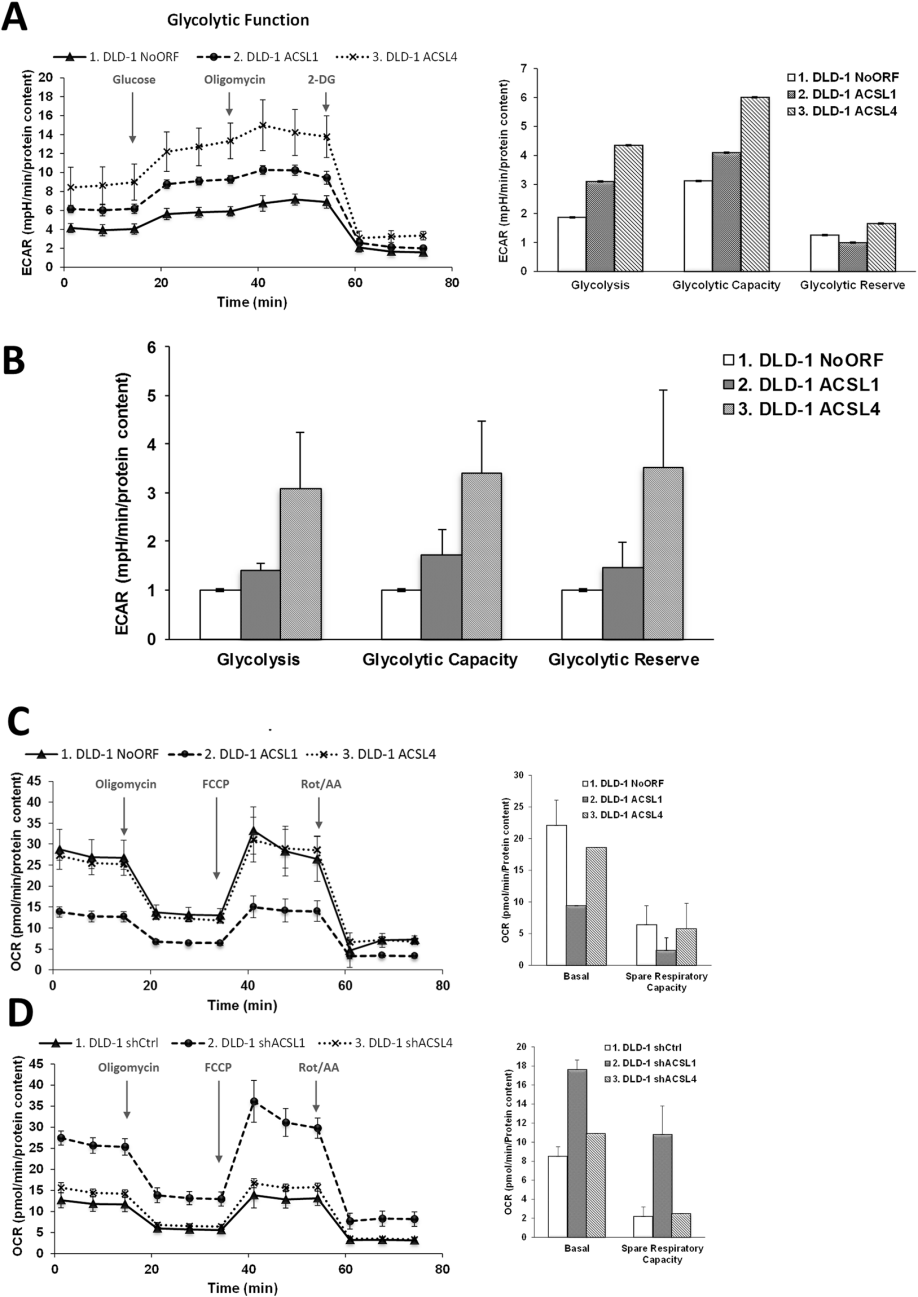
Bioenergetic characterization of ACSL1 and ACSL4 isoforms. (**A**) Glycolytic profile of No ORF, ACSL1 and ACSL4 cells. Cells were starved 1 h and a half and Extracellular Acidification Rate (ECAR) levels were measured using a Seahorse extracellular flux analyser. Addition of 10 µM glucose was used for the measurement of glycolysis rate. Maximal ECAR was measured upon 0.5 µM Oligomycin treatment. 50 mM 2-DG inhibits all glycolysis showing non-glycolytic acidification. Right panel shows the quantification of Glycolysis (measurement of glycolytic process rate), Glycolytic Capacity (Maximum response to glycolytic demand from stress) and Glycolytic Reserve (Reserve capacity available to utilize glycolysis beyond the basal rate). (**B**) Average quantification of glycolytic parameters in 3 independent experiments (n = 3) with 12 replicates each. (**C**) Oxygen consumption rate (OCR) of No ORF, ACSL1 and ACSL4 cells. Bioenergetics parameters were obtained by adding 2 µM Oligomycin to block ATP-linked OCR, 0.2 µM FCCP to uncouple mitochondria for maximal OCR and 0.5 µM Rotenone/Antimycin A (Rot/AA) to shut down mitochondrial respiration. Right panel reflects the quantification of basal respiration (oxygen consumption used to meet cellular ATP demand, calculated by subtracting non-mitochondrial OCR obtained upon Rot/AA addition) and spare respiratory capacity (capability to respond to an energetic demand, calculated as the difference between maximal and basal OCR). (**D**) OCR measurements over time for cells stably expressing shRNAs for ACSL1 (shACSL1), ACSL4 (shACSL4) or scramble (shCtrl) and respiratory parameters quantification (right panel). **A**, **C** and **D** show representative experiments of 3 or 4 experiments (n = 3).

It is well know the relation between metabolic alterations fuelling different malignant aspects of tumour cells, such as the pro-proliferative Warburg effect^42^. For this reason, we wanted to check the glycolytic potential, as the aerobic glycolysis is one of the most remarkable features of proliferative cancer cells. Extracellular acidification rate was measured to assay glycolytic function of ACSL1, ACSL4 and No ORF control DLD-1 cells. In accordance with their proliferative potential, the highest basal glycolysis, glycolytic capacity and reserve was found for ACSL4 cells when compared to No ORF cells, finding an intermediate glycolytic phenotype for ACSL1 cells (Fig. 2A,B). We further wanted to check the oxidative metabolism reflecting the mitochondrial activity of these cell lines (Fig. 2C). Differently, ACSL4 presented both, a basal and maximal oxygen consumption rate (OCR) similar to No ORF cells, however, ACSL1 basal OCR was significantly lower than the other cell clones, highlighting again the metabolic differences among ACSL isoforms. Consequently, knocking down of ACSLs by shRNA means presented the opposite effect. In this case, shACSL1 cells presented the highest basal and maximal OCR, and again, ACSL4 and control (shCtrl) cells presented a parallel behaviour (Fig. 2D). All these data suggest that different metabolic performances are associated with distinctive tumorigenic features in cancer cells.

### Metabolic profiling segregates ACSL isoforms and malignant features of CRC cells

**Figure 3:**
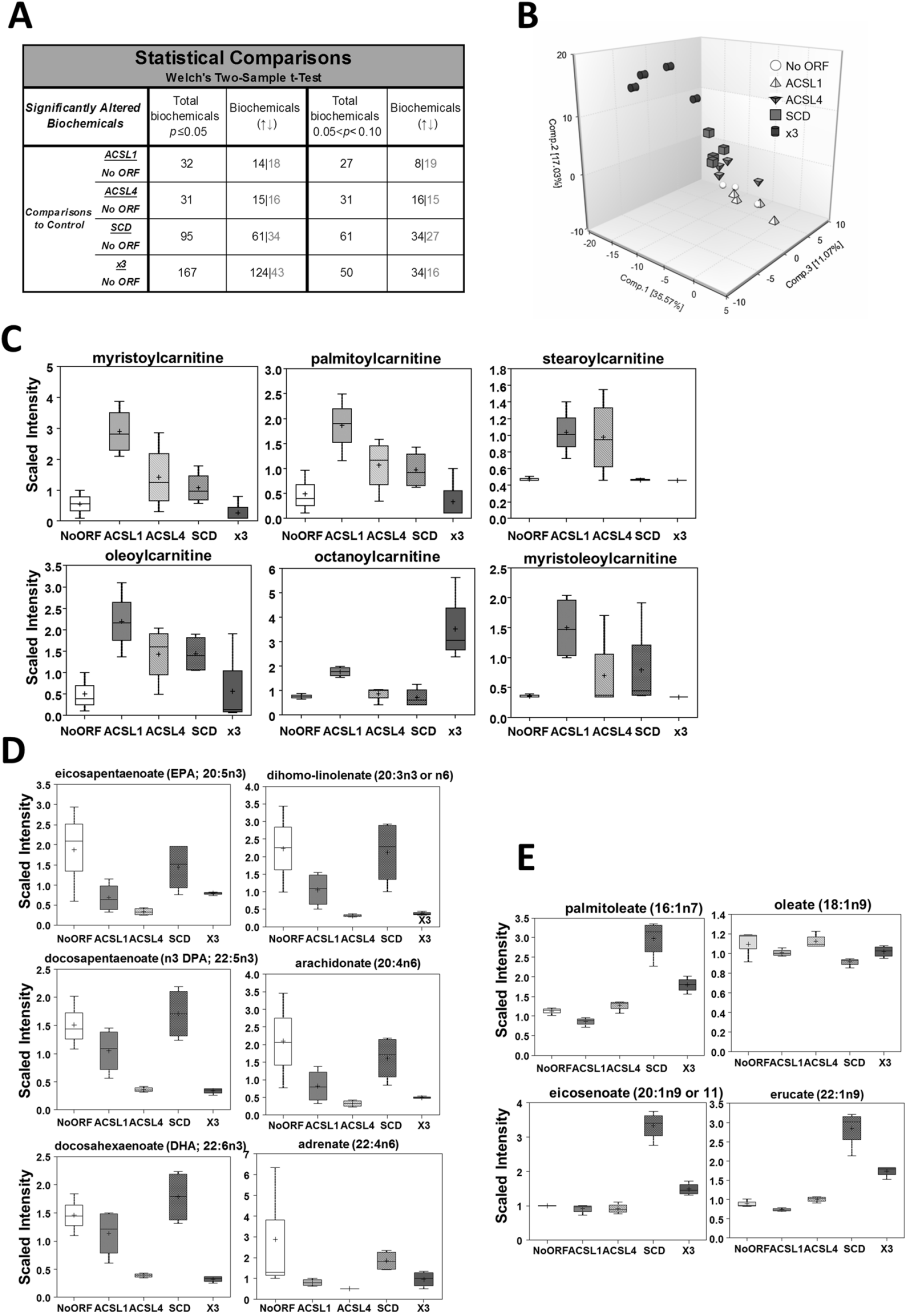
Individual overexpression of ACSL1, ACSL4 or SCD associates to changes in specific sets of metabolites. (﻿**A**﻿) Metabolomic analysis statistical summary of colon cancer cells overexpressing ACSL1, ACSL4 and SCD. Table indicates the number of biochemicals with statistical significance (p ≤ 0.05) or with approaching significance (0.05 < p < 0.10) from dataset analysis with a total 494 named biochemicals detected. Black numbers indicate upregulated metabolites as grey colour indicates the downregulated ones. Welch’s two-sample t-test was used to identify biochemicals that differed significantly between experimental groups. (**B**) Principal component analysis (PCA) segregation of DLD-1 expression-subtypes based on differences in metabolic signature. (**C**) Acylcarnitines are elevated most dramatically in DLD-1 cells expressing ACSL1. Box plots show the scaled intensity (Y axis) for each chemical. Mean, median and maximum and minimum values of the distribution are represented in the plots. (**D**) DLD-1 cells expressing ACSL4 present lower PUFA levels. Box plots for the levels of representative polyunsaturated fatty acids are shown. (**E**) Monounsaturated fatty acids are elevated in DLD-1 cells expressing SCD. Levels of the unsaturated fatty acids palmitoleate, oleate, eicosenoate and erucate are represented.

To gain further insight into the functional differences between ACSL1 and ACSL4, and to elucidate the metabolic features corresponding to the ACSL/SCD network enzymes, we performed metabolomics analysis using ACSL1 and ACSL4 cells together with the corresponding DLD-1 cells overexpressing SCD (SCD cells) and the three enzymes at the same time (x3 cells)^19^. A total of 494 differential biochemicals were identified. Figure 3A shows a summary of the numbers of metabolites that achieved statistical significance (p ≤ 0.05), as well as those with approaching significance (0.05 < p < 0.10). A principal component analysis, (PCA), was done to visualize how samples, within a group, cluster with respect to their data-compressed “principle components”. Figure 3B shows how all sample groups were well segregated from each other based on differences in their overall metabolic signature. According to this analysis, ACSL1 samples were the ones that were less separated from No ORF control ones, and, thus, more similar regarding their metabolic profiles. In contrast, the combination of overexpressed enzymes (x3) generated an overall metabolite profile most significantly diverged from the control.

Regarding the individual metabolic characteristics for each enzyme overexpression, ACSL1 cells were mostly characterized by an increase in acylcarnitine levels (Fig. 3C, Supplementary File 1). While ACSL4 and SCD cells also displayed some increases in acylcarnitines relative to control, ACSL1 was consistently the highest. Acylcarnitines are generated through the transfer of carnitine for CoA on acyl-CoA derivatives of long-chain FA by carnitine palmityoltransferase (CPT), to transport them through the mitochondrial membrane. Thus, elevated acylcarnitine levels can be due to increased CPT activity resulting from an increase in the cytoplasmic acyl-CoA substrate levels, such as the ACSL1 products.

In spite of belonging to the same family that ACSL1 and the possibility to perform equivalent reactions, lower levels of polyunsaturated fatty acids (PUFA) were the most remarkable feature of ACSL4 cells (Fig. 3D, Supplementary File 1). Not surprisingly, among the significantly downregulated PUFA we can find all preferred ACSL4 substrates, such as arachidonate, docohexanoate and eicosapentaenoate. ACSL1 cells presented downregulated PUFA levels as well, even though it was not so marked as in ACSL4 cells, suggesting certain function overlapping.

As expected, several monounsaturated fatty acids (MUFA) were elevated relative to control in the SCD overexpressing cells, while their corresponding saturated substrate fatty acids were not (Fig. 3E, Supplementary File 1). Palmitoleate, one of the main products of SCD was clearly upregulated in these cells. Even though we did not find an elevation of the enzyme main product, oleate (18:1n9), this can be explained by conversion into eicosenoate (20:1n9) and erucate (22:1n9) upon elongase action.

**Figure 4:**
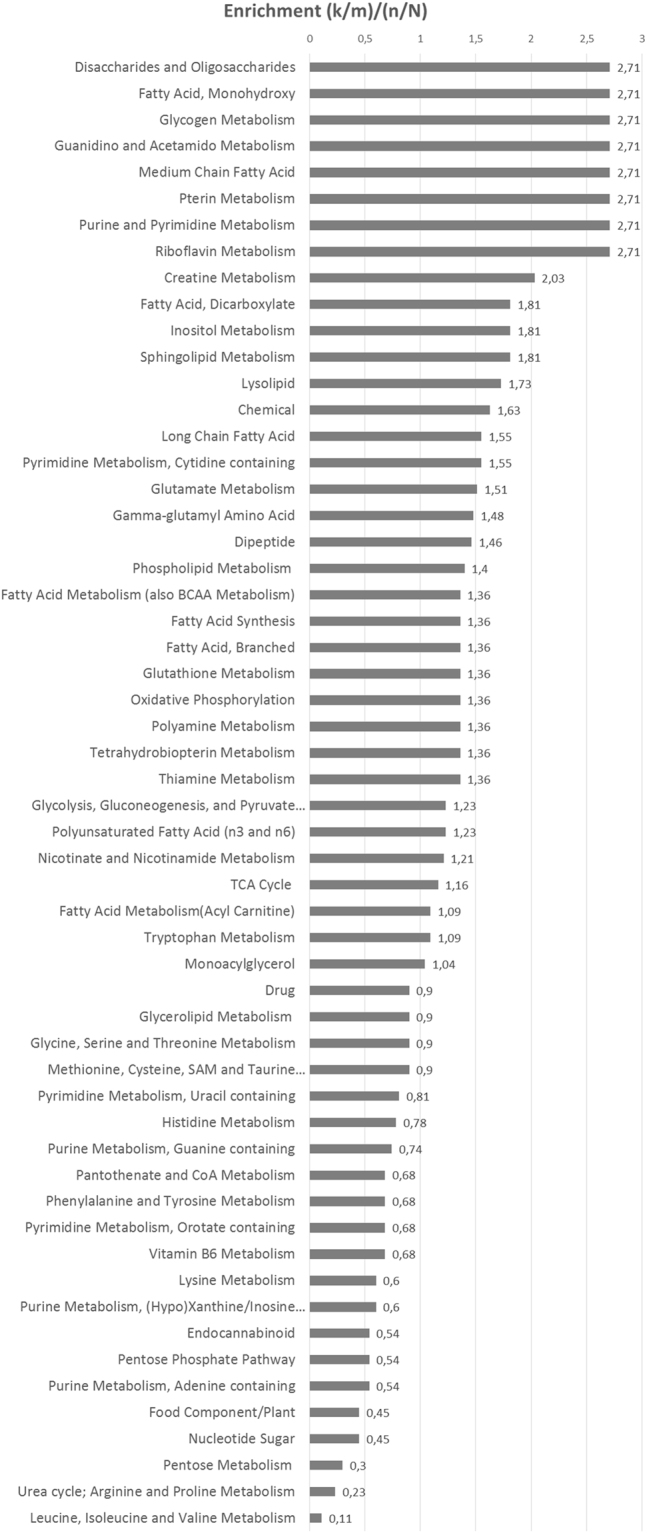
Pathway enrichment of cells simultaneously overexpressing ACSL1, ACSL4 and SCD. Plot shows the main metabolic pathways differentially regulated upon ACSL1, ACSL4 and SCD overexpression (x3).

**Figure 5:**
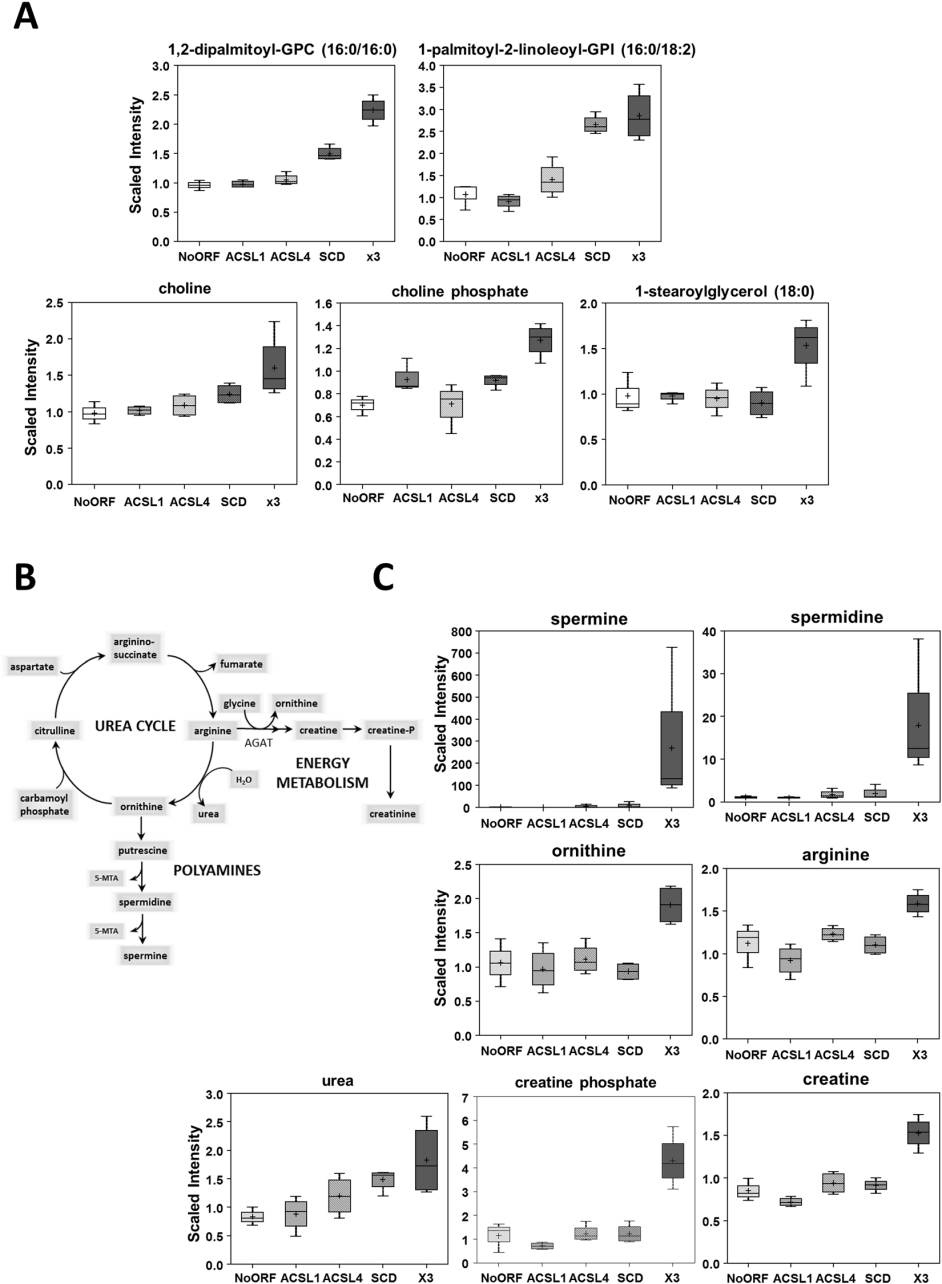
Increased phospholipids levels and urea cycle metabolites characterize x3 cells. (**A**) Higher phospholipid levels in DLD-1 cells expressing ACSL1, ACSL4 and SCD along with increased levels of choline, choline phosphate and monoacylglycerols. GPC = Glycerophosphocholine, GPI = glycosylphosphatidylinositol. (**B**) Schematic view of urea cycle and derived routes. (**C**) Urea Cycle-derived metabolites are highly elevated in x3 cells. The upregulated levels in x3 cells of the polyamines spermine and spermidine, the urea cycle intermediates ornithine and arginine, as well as the ones for creatine and creatine phosphate are presented. Box plots show the scaled intensity (Y axis) for each metabolite. Mean, median, and maximum and minimum values of the distribution are represented.

The most striking results were found for the x3 cells. Figure 4 shows the main metabolic pathways enriched upon ACSL1, ACSL4 and SCD simultaneous overexpression, mainly involved in fatty acid, carbohydrate, nucleotides and energy metabolism. As a first distinctive feature, x3 cells showed higher levels of many phospholipids in the phosphatidylcholine, phosphatidylethanolamine and phosphatidylinositol classes (Fig. 5A, Supplementary File 1). Phospholipids make up the largest lipid component of cell membranes, crucial for cell proliferation and cancer signalling. x3 cells also displayed elevated choline and choline phosphate levels (Fig. 5A) that could be a result of phospholipid degradation, but lysolipids are generally down in the SCD and x3 cells, consistent with a decrease in phospholipid turnover (Supplementary File 1). In addition, many monoacylglycerols were up in x3 as well as in SCD relative to No ORF cells (Fig. 5A, Supplementary File 1). Other lipids varied in an opposite sense, such as sphinganine whose levels drop precipitously in x3 cells while, accordingly, products downstream of sphinganine were elevated, such as phosphoethanolamine, the ceramide N-palmitoyl-sphingosine and sphingomyelin (Supplementary Figure 2 and Supplementary File 1).

The second distinctive feature of ACSL/SCD axis was the elevation of some urea cycle derived metabolites (Fig. 5B). The highly-elevated polyamines spermine and spermidine derive from the urea cycle metabolite ornithine and other urea cycle metabolites such as arginine, ornithine and urea were also elevated (Fig. 5C). Elevated polyamine levels have been associated with increased cell proliferation and other malignant features^43,44^. Moreover, creatine and creatine phosphate were also elevated in x3 cells, potentially indicating a more favourable energy status in those cells relative to the other clones (Fig. 5C).

The glycolysis pathway was also altered in x3 cells. The intermediates dihydroxyacetone phosphate, 3-phosphoglycerate and phosphoenolpyruvate (PEP) were strongly elevated in x3 cells (Supplementary Figure 3 and Supplementary File 1). PEP levels elevation could be due to a decline in pyruvate kinase (PK), or an increase in phosphofructosekinase-1 (PFK-1) activities. Despite the much higher PEP levels, pyruvate levels were not significantly changed. PK activity can be down-regulated by increases in Acetyl-CoA levels, coincident with higher Acetyl-CoA in x3 cells. PFK-1 increased activity is also supported by decreased levels of the PFK-1 substrate, fructose-6-phosphate and elevation of its product, fructose 1,6-bisphosphate, up in x3 cells (Supplementary Figure 3 and Supplementary File 1).

**Figure 6:**
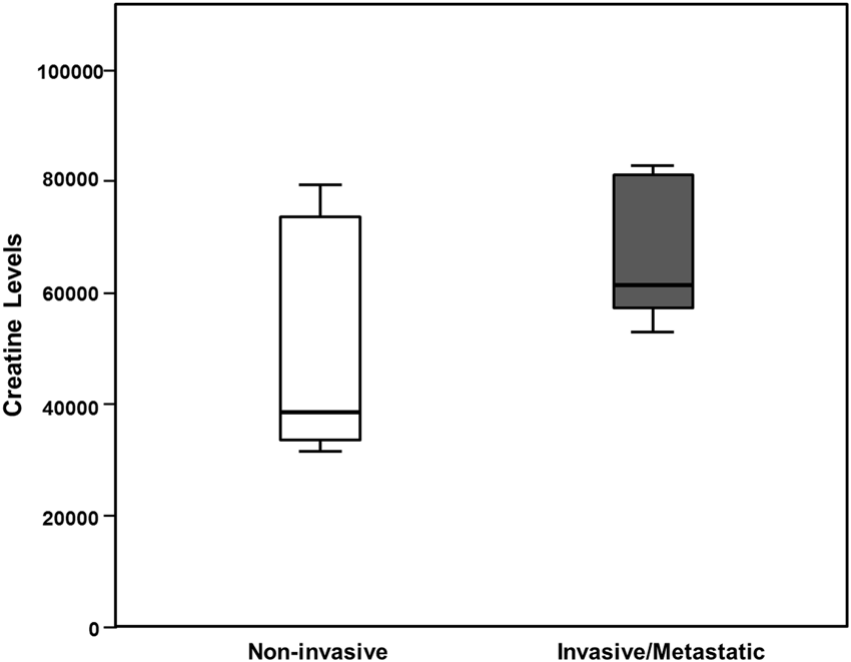
The more invasive and metastatic CRC cells display increased creatine levels. Correlation of Creatine levels with invasiveness in different CRC cell lines. Non-invasive cell lines: DLD-1 NoORF, HCT116, HT29, CaCo2, LS174T and SW480. Invasive or metastatic cell lines: DLD-1 x3, SW620, Colo205, LoVo and T84.

Increases in the amount of phospholipids, sphingomyelins and ceramides and polyamines, have been reported to stimulate proliferation^23,44^. Thus, the more aggressive characteristics of x3 cells could be due to an increased cell proliferation. We have previously reported that the simultaneous overexpression of ACSL/SCD was not accompanied of increased proliferation^19^. Figure 1A shows how ACSL1/ACSL4/SCD simultaneous overexpression (x3 cells) not only does not increase cell proliferation but also causes the opposite effect, presenting the lowest proliferation rate among all the cell lines. Thus, x3 cells altered metabolic profile should be fuelling malignant characteristics other than proliferation, as invasiveness or a favoured energetic balance. In this sense, more invasive and metastatic CRC cell lines presented increased creatine levels compared with less invasive and primary tumour derived cells (Fig. 6). Hence, upregulated creatine pathway (Figs 4, 5C and 6) could reflect a more favourable energy status that could be crucial for processes other than cell proliferation such as cell invasion.

### ACSL/SCD overexpression phenocopies metabolic features of metastatic cells

**Figure 7:**
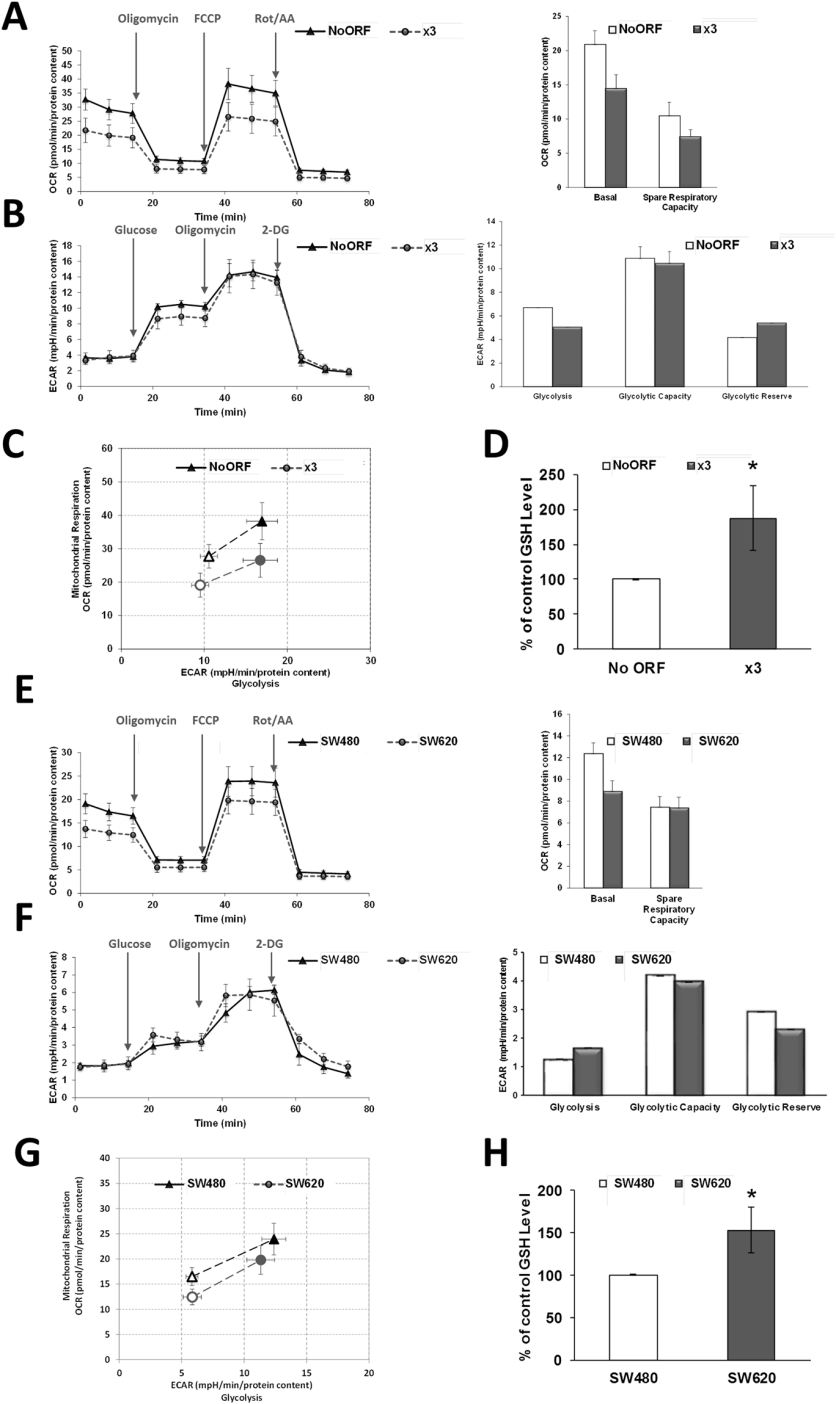
Bioenergetic profile of cells overexpressing ACSL1, ACSL4 and SCD resembles those from metastatic cells. (**A**) Seahorse assay of oxygen consumption rate for No ORF and x3 cells before and after 1 µM Oligomycin, 0.2 µM FCCP and 0.5 µM Rotenone/Antimycin A (Rot/AA) addition. Basal respiration and spare respiratory capacity quantification (right panel). (**B**) No ORF and x3 cells glycolytic profile. Cells were starved 1 h and a half and ECAR levels were measured before and after addition of 10 µM glucose, 0.5 µM Oligomycin and 50 mM 2-DG. Right panel shows the quantification of Glycolysis, Glycolytic Capacity and Glycolytic Reserve for each DLD-1 clone. (**C**) Metabolic phenotype plot depicting the relative metabolic state of cells under baseline and stressed conditions. Baseline Phenotype: OCR and ECAR of cells at starting assay conditions (empty circle and empty triangle). Stressed Phenotype: OCR and ECAR of cells under an induced energy demand, here achieved upon FCCP treatment (full circle and full triangle). (**D**) Levels of reduced glutathione for DLD-1 No ORF and x3 cells. (**E**) Seahorse assay of oxygen consumption rate for SW480 and SW620 cells before and after 1 µM Oligomycin, 0.6 µM FCCP and 0.5 µM Rot/AA addition. Quantification of basal respiration and spare respiratory capacity (right panel). (**F**) Glycolytic profile of SW480 and SW620 cells. Cells were starved 1 h and a half and ECAR levels were measured before and after addition of 10 µM glucose, 0.5 µM Oligomycin and 50 mM 2-DG. Right panel shows the quantification of Glycolysis, Glycolytic Capacity and Glycolytic Reserve for each cell type. (**G**) Metabolic phenotype plot depicting the relative metabolic state of cells under baseline and stressed conditions. Baseline Phenotype: OCR and ECAR of cells at starting assay conditions (empty circle and empty triangle). Stressed Phenotype: OCR and ECAR of cells under an induced energy demand, such as FCCP treatment (full circle and full triangle). (**H**) Levels of reduced glutathione for SW480 and metastatic SW620 cells. (**A**,**B**,**C**,**E**,**F** and **G**) show representative experiments of 3 or more experiments (n = 3). Results in (**D** and **H**) represent the mean ± SD of 3 experiments (n = 3). *p < 0.05.

This improved energetic status was further studied by analysing OCR in x3 cells. This invasive cell line presented a decreased basal OCR when compared with non-invasive No ORF cells (Fig. 7A), that, consequently, derived in a decreased OCR consumption under stressed conditions (FCCP addition). However, differences were not so patent when glycolysis was assayed, since extracellular acidification rate (ECAR) was mostly similar (or slightly less) to control cells (Fig. 7B). When both parameters of cell metabolism were analysed in a combined way, without main differences in glycolysis, the core dissimilarity was a lesser basal rely of oxidative metabolism in mitochondria (Fig. 7C). This attenuated basal oxidative metabolism is in accordance with less oxidative stress in x3 cells, as is reflected by increased reduced glutathione (GSH) levels in x3 cells (Fig. 7D) and upregulated general glutathione metabolism (Fig. 4 and Supplementary File 1).

We wondered if these metabolic differences identified in ACSL/SCD overexpressing cells were a common feature of other invasive cells. For this purpose, we comparatively studied metabolic differences among primary tumour SW480 cells and SW620 cells, derived from a metastasis of the same patient. Interestingly, the metastatic cell line presented a lower basal respiration (Fig. 7E) compared to the parental SW480 cell line, equivalent to the difference found among respiratory behaviour of x3 cells when compared with their non-invasive control No ORF cells. Similarly, there were no substantial differences in glycolytic performance between SW480 and SW620 cells (Fig. 7F), and again, we can conclude that the major difference among primary tumour and metastatic CRC cells was that they can maintain their energetic homeostasis with a lower basal rate of mitochondrial oxidation without glycolytic changes (Fig. 7G). Likewise, increased levels of GSH were also found for metastatic SW620 cells, very likely indicating a lower oxidative stress (Fig. 7H).

## Discussion

In this work, we have analysed the metabolic characteristics defining ACSL/SCD action on CRC cells and their connection with the protumorigenic features intrinsic to each enzyme network integrant, highlighting the functional dissimilarities among the ACSL members that demanded further attention. ACSL1 and ACSL4 showed an association between high expression of each isoform and poorer clinical outcome of stage-II CRC patients that resulted in a stronger and more potent association with patient relapse in the context of the ACSL/SCD network^19^. Besides, systematic analysis of ACSLs expression and clinical outcomes in several human cancers points towards ACSL1 upregulation in CRC and breast cancer and downregulation in lung cancer. Similarly, ACSL4 has been found upregulated in CRC, but in contrast predicted better prognosis in breast, brain and lung tumours. Therefore, ACSLs member’s role in the development of different types of tumours can be diverse, finding even ACSLs with a tumour suppressor profile such as ACSL5 and ACSL6^45^.

Our results show that ACSL4 preferentially stimulates proliferation in CRC cells (Fig. 1A) and this is associated to a more glycolytic phenotype compared to control or ACSL1 cells, without major changes in mitochondrial performance (Fig. 2A, 2B and 2C). In contrast, ACSL1 is characterized by minor effects in proliferation or glycolytic performance (Figs 1A and 2A), though presents an inclination to invasive capabilities accompanied by a decreased basal OCR (Figs 1D,E and 2C) as found for the invasive x3 cells. This was further validated when opposite results were found upon the use of specific shRNAs (Figs 1B and 2D). In similar fashion, metabolic profiles were substantially different for both ACSL isoforms, highlighting the functional differences among them.

**ACSL1** overexpression was mostly characterized by the elevation of acylcarnitines probably owed to increased CPT activity upon augmented acyl-CoA levels caused by ACSL1 overexpression (Fig. 3C). For instance, elevated oleoylcarnitine agrees with the fact that oleate is one of the ACSL1 preferred substrates. Upregulated acylcarnitines could be also associated with a fatty acid oxidation (FAO) reduction in the mitochondria that should be accompanied by decreased Acetyl-CoA, down slightly in ACSL1, but up significantly in x3 cells (Supplementary Figure 3). This could be connected to the lower basal OCR registered for ACSL1 cells (Fig. 2C) but not for the lower basal OCR in x3 (Fig. 7A). Nevertheless, acylcarnitines might be mediating invasive capabilities important for cancer progression since these FAO intermediates levels have been found dramatically upregulated in several malignancies^46–49^.

Lower PUFA were the defining feature of **ACSL4** cells. This may reflect greater PUFA utilization to synthesize complex lipids, such as the phospholipids required for membrane biogenesis, crucial for cancer cells. Though elevated phospholipids were not characteristic of ACSL4 cells (Supplementary File 1), this could be the key contribution of ACSL4 to the ACSL/SCD network phenotype as demonstrated by elevated phospholipids levels in x3 cells (Fig. 5A). In line with this, it has been described in other cell contexts ACSL4-mediated PUFA incorporation into complex lipids^50,51^. Not surprisingly, **SCD** desaturase overexpression was accompanied by a clear MUFA upregulation, its natural products. Curiously, most of these MUFAs were lower in x3, which may again reflect greater incorporation into complex lipids. In general, SCD cells metabolic profile is the most similar to x3 cells and they present similar proliferation tendencies as ACSL/SCD cells though with a mild effect (Fig. 1A,B and Supplementary File 1). This is in agreement with previous findings in which SCD overexpression induced Erk and Akt activation, as was also the case with x3 cells^19^. Nonetheless, a complete EMT and invasive phenotype is only found in the case of the full ACSL/SCD axis overexpression, though it has been described a SCD knock-down impairment of EMT-like behaviour in other tumours^52^. Together with ACSL1 fuelling the mitochondria and its pro-invasive actions, and ACSL4 PUFA managing for complex lipid formation, SCD could help providing an appropriate MUFA/PUFA ratio that could stimulate further invasive signalling mediated by increased levels of phospholipids and ceramides in ACSL/SCD cells. In this sense, unsaturated fatty acids seem to be crucial for ACSL/SCD phenotype.

Interestingly, x3 cells metabolic landscape cannot be explained as the sum of ACSL1, ACSL4 and SCD individual profiles. Unexpectedly, x3 cells displayed upregulated complex lipids such as phospholipids and sphinganine-derived ceramides and sphingolypids (Figs 4 and 5A and Supplementary Figure 2) which through lipid signalling may alter key regulatory pathways^21–23^ such as Akt, activated in x3 cells^19^. Furthermore, affected pathways not directly associated with lipid metabolism, such as urea cycle and glycolysis were found (Fig. 5B,C and Supplementary Figure 3). The urea cycle stimulation could be derived from the increased Acetyl-CoA levels that x3 cells present (Supplementary Figure 3). Polyamines have been extensively related to cell proliferation and growth^43,44^. Recently, they have been involved as crucial for protein translation, key to cancer invasion and metastasis^53,54^. Inhibition of polyamine synthesis also decreased the amount of metastasis in several cancer models^44^. Polyamines could be distinctive oncometabolites of metastatic cells, since metastatic SW620 cell present higher levels of polyamines than their corresponding primary tumour cells, SW480 as well as higher activity of the key regulatory enzymes of polyamine biosynthesis^55^. Creatine and phosphocreatine were also clearly upregulated in x3 cells (Figs 4 and 5C) indicating a more advantageous energetic status that could be essential for invasive features development. In this sense, phosphocreatine, has been reported to directly fuel tumour growth and liver metastasis in CRC^56^, and both creatine and creatinine downregulate toll-like receptors (TLRs) expression in macrophages^57^, which could be used by tumour cells as an immunosuppressive strategy to favour metastasis. This is in accordance with our results showing increased creatine levels in the more invasive and metastatic CRC cells (Fig. 6). Regarding glycolytic perturbations (Supplementary Figure 3), increased PEP levels and normal pyruvate could be a reflect of less demand of TCA feeding from pyruvate (from carbohydrates) explaining lower basal OCR consumption since a more energetic status is achieved through other alternative supplies, such as FAO, that could be fed by ACSL1 overexpression. Nevertheless, FAO inhibitor etomoxir is not sufficient to revert the EMT phenotype of ACSL/SCD cells that, conversely, can be achieved upon a more drastic energetic restriction caused by AMPK signalling reactivation upon metformin treatment^19^ (Supplementary Figure 4). Thus, it seems that x3 cells present a better overall energetic status that even though it is not due to observable differences in ATP content (data not shown), is reflected by lower basal OCR and elevated creatine pathway that could be partly fed by ACSL1-drived increased FAO and sustained through other levels dependent on further phospholipid signalling supported by ACSL4 and SCD increased activities as PUFA and MUFA suppliers, respectively.

Importantly, x3 cells metabolic capacities were reproduced in a model of primary and metastatic CRC cell lines, SW480 and SW620. More invasive or metastatic cells, performed mitochondrial oxidation at a lower rate without glycolytic changes, pointing to an overall energetic advantage in invasive cells that could derive from increased energetic efficiency or from an augmented utilization of alternative fuels. Furthermore, both invasive x3 and metastatic SW620 cells presented increased GSH compared to their corresponding non-invasive control cells (Fig. 7D,H). This lower oxidative stress is further supported by increased gammaglutamyl-aminoacids in x3 cells (Supplementary File 1), as markers of glutathione regeneration and decreased levels of NADPH (Supplementary File 1), which could be explained by greater use for GSH generation, since pentose phosphate pathway presented no downregulation (Supplementary File 1). Furthermore, the other core reducing agent, NADH presented upregulated levels in x3 cells. Although oxidative stress role in tumour progression has been controversial, new studies indicate that cancer cells must increase their capacity to withstand oxidative stress to produce distant metastasis^58–60^.

## Methods

### Cell culture, stable cell lines generation and reagents

Cell lines, obtained from ATCC (ATCC, Manassas, VA, USA) were cultured in DMEM 10% FBS and maintained under standard conditions. Etomoxir and Metformin were purchased form Sigma-Aldrich (Sigma-Aldrich, St. Louis, MO, USA). Images were captured using a Leica DM IL microscope (Leica Microsystems, Wetzlar, Germany), with a 10X Plan Fluotar objective and registered using Leica Application Suite (LAS). DLD-1 cells stably overexpressing ACSL1, ACSL4, SCD and ACSL1/ACSL4/SCD (x3) were generated with specific lentivirus or an equivalent control vector which does not express any ORF (No ORF cell line) as described^19^. For shACSL1, shACSL4 and shSCD cells generation, HEK 293T cells were transfected with Mission specific lentiviral vectors (TRCN0000045518, TRCN0000045541, TRCN0000312672) or a shControl pLKO.1 empty vector (Sigma-Aldrich, St. Louis, MO, USA) along with packaging plasmids (Addgene, Cambridge MA, USA). Supernatant produced upon 48 h transfection in HEK293T cells was used to infect DLD-1 cells followed by puromycin selection (2 µg/ml) during 1 week.

### Quantitative real-time PCR

RNA (400 ng) was reverse-transcribed using the High Capacity RNA-to-cDNA Master Mix system (Life Technologies, Carlsbad CA, USA). qPCR was performed using VeriQuest SYBR Green qPCR Master Mix (Affymetrix, Santa Clara, CA, USA) in the 7900HT Real-Time PCR System (Life Technologies). Gene specific primers for *ACSL1* (Fw: 5′-ACATTATGTTCCTGGGCCCA-3′ and Rv: 5′-AGTCAGAAGGCCATTGTCGA-3′), *ACSL4* (Fw: 5′-GGCACAACAGAAAGGGGTAG-3′ and Rv: 5′-GGTTCCTCAGCTCCTTCCTT-3′), *SCD* (Fw: 5′-TGCCCACCACAAGTTTTCAG-3′ and Rv: 5′-CATCAGCAAGCCAGGTTTGT-3′), *CDH2* (N-cadherin) (Fw: 5′-CGGTTTCATTTGAGGGCACA-3′ and Rv: 5′-TTGGAGCCTGAGACACGATT-3′) and SNAI2 (Slug) (Fw: 5′-CGTTTTCCAGACCCTGGTT-3′ and Rv: 5′-CTGCAGATGAGCCCTCAGA-3′) were used and *GAPDH* expression (Fw: 5′-TGGTATCGTGGAAGGACTCATGAC-3′ and Rv: 5′-ATGCCAGTGAGCTTCCCGTTCAGC-3′) used for normalization. Relative gene expression was calculated using the 2−∆∆Ct method.

### Western Blot and antibodies

Cells were lysed in Laemmli buffer and boiled at 95 °C for 10 min. Proteins were separated by SDS–polyacrylamide gel electrophoresis and transferred onto a nitrocellulose membrane (Bio-Rad Laboratories, Hercules, CA, USA). The membranes were blocked using 5% non-fat dry milk in TBS 0.05% Tween-20, and incubated with primary antibodies overnight at 4 °C. After incubation with secondary antibodies, Clarity Western ECL Substrate (Bio-Rad Laboratories) was used for signal detection and Vinculin determination or unspecific band were used as loading controls. Anti-human ACSL4 was generously provided by Dr. Stephen Prescott, University of Utah, Salt Lake, USA and Dr. Diana Stafforini, Huntsman Cancer Institute, University of Utah, USA, and used as indicated^61^. Antibody against SCD^62^ was a kind gift from Dr. Jean-Baptiste Demoulin, Université Catholique de Louvain, Brussels, Belgium. anti ACSL1 (4047) was obtained from Cell Signaling (Cell Signaling Technology Inc., Beverly, MA, USA) and anti-Vinculin (V9131) and β-Actin (A1978) were from Sigma.

### Proliferation assays

Proliferation was analysed in real-time using the xCELLigence™ system (ACEA Biosciences, San Diego, CA). Real-time monitoring of cell proliferation, xCELLigence™ system measurements were performed by spreading 10000 cells over a FN-coated gold electrode sensor plate. Cellular impedance recordings converted to a cell index (CI) allow for the assessment of attached cells. Real-time monitoring of proliferation was performed for 8 days in 15 min intervals.

### Wound healing

A density of 40000 cells per reservoir was plated using IBIDI-Inserts (IBIDI GmbH, München, Germany) and incubated until confluence was reached. Upon inserts removal, migration was monitored and registered every 12 h using a 10X Plan Fluotar objective (Leica).

### Invasion assays

BD Matrigel^TM^ invasion chambers (BD Biosciences) were seeded with 50000 cells in serum-free DMEM. After 48 h, using DMEM 10% FBS as a chemoattractant, inserts were fixed and stained with crystal violet. Once non-migrated cells were removed with cotton swaps, pictures were taken using an Olympus CKX41 microscope (Olympus, Tokyo, Japan), with a 20X LCAch objective and registered using analysis getIT software (Olympus).

### Oxygen consumption rate (OCR) and extracellular acidification rate (ECAR)

OCR and ECAR were monitored as indicators of mitochondrial respiration and glycolytic function study with an XF96 Extracellular Flux Analyzer using XF Cell Mito Stress Test kit and XF Glycolysis Stress kit according to manufacturer instructions (Seahorse Biosciences, North Billerica, MA, USA). Cell seeding number was optimized (50000 cells/well for DLD-1 and SW480 cells and 90000 cells/well for SW620 cells. For mitochondrial stress test, cells were plated into XF96 plates and regular culture media was replaced at 24 hours with 2 mM pyruvate, 2 mM glutamine and 10mMglucose supplemented Base media (Seahorse Bioscience) upon several washes. Cells were placed in a non-CO_2_ 37 °C incubator for 1 hour, prior to assay. Upon basal rate measurements were taken, mitochondrial respiratory chain drugs were added, following Mito Stress kit specifications. 2 µM Oligomycin was used to block ATP-linked oxygen consumption, 0.2 µM FCCP (0.6 µM for SW480 and SW620 cells) as an uncoupling agent to obtain maximal respiration and 0.5 µM Rotenone/Antimycin A to inhibit complex I and III, stopping all mitochondrial respiration. For glycolysis analysis, 2 mM pyruvate and 2 mM glutamine supplemented Base media was used and cells were incubated without CO_2_ for 1 hour and a half. Following Glycolysis Stress kit specifications, 10 µM glucose was injected to stimulate glycolysis, 0.5 µM Oligomycin to obtain maximal glycolytic capacity upon oxygen consumption inhibition and 50 mM 2-Deoxy-D-glucose (2-DG) to shut down all glycolysis. OCR and ECAR were measured 3 times following injection of each drug, and normalized to protein content. At least 6 replicates per condition were done for each experiment.

### Statistical analysis

Significance between groups was determined by t-test. All reported p values were two-sided. Statistical significance was defined as p < 0.05. The statistical analyses were performed using the R statistical software version 3.1.1 (www.r-project.org).

### Global metabolomic profiling

Eleven million of cells from each DLD-1 cell clone were collected, rinsed with PBS, and the snap-frozen cell pellets were submitted to Metabolon Inc for global metabolomic analysis. Each condition included four replicates. A combination of GC-MS and LC-MS methods were used, and each metabolite amount was normalized to total protein amount of the individual cell pellets. Briefly, proteins were precipitated with methanol under vigorous shaking for 2 min (Glen Mills GenoGrinder 2000) followed by centrifugation to recover chemically diverse metabolites. The UPLC-MS/MS portion was based on a Waters ACQUITY ultra-performance liquid chromatography (UPLC) and a Thermo Scientific Q-Exactive high resolution/accurate mass spectrometer interfaced with a heated electrospray ionization (HESI-II) source and Orbitrap mass analyser operated at 35,000 mass resolution. The sample extract was dried then reconstituted in acidic or basic LC-compatible solvents, each of which contained 8 or more injection standards at fixed concentrations to ensure injection and chromatographic consistency. One aliquot was analysed using acidic positive ion optimized conditions and the other using basic negative ion optimized conditions in two independent injections using separate dedicated columns (Waters UPLC BEH C18–2.1 × 100 mm, 1.7 µm). Extracts reconstituted in acidic conditions were gradient eluted from a C18 column using water and methanol containing 0.1% formic acid. The basic extracts were similarly eluted from C18 using methanol and water, however with 6.5 mM Ammonium Bicarbonate. The third aliquot was analysed via negative ionization following elution from a HILIC column (Waters UPLC BEH Amide 2.1 × 150 mm, 1.7 µm) using a gradient consisting of water and acetonitrile with 10 mM Ammonium Formate. The MS analysis alternated between MS and data-dependent MS2 scans using dynamic exclusion, and the scan range was from 80–1000 m/z. The samples destined for analysis by GC-MS were dried under vacuum for a minimum of 18 h prior to being derivatized under dried nitrogen using bistrimethyl-silyltrifluoroacetamide. Derivatized samples were separated on a 5% diphenyl / 95% dimethyl polysiloxane fused silica column (20 m × 0.18 mm ID; 0.18 um film thickness) with helium as carrier gas and a temperature ramp from 60° to 340 °C in a 17.5 min period. Samples were analysed on a Thermo-Finnigan Trace DSQ fast-scanning single-quadrupole mass spectrometer using electron impact ionization (EI) and operated at unit mass resolving power. The scan range was from 50–750 m/z. Raw data was extracted, peak-identified and QC processed using Metabolon’s hardware and software. Compounds were identified by comparison to library entries of purified standards or recurrent unknown entities.

### Principal Components Analysis (PCA)

Principal components analysis is an unsupervised analysis that reduces the dimension of the data. Each principal component is a linear combination of every metabolite and the principal components are uncorrelated. The number of principal components is equal to the number of observations. The first principal component is computed by determining the coefficients of the metabolites that maximizes the variance of the linear combination. The second component finds the coefficients that maximize the variance with the condition that the second component is orthogonal to the first. The third component is orthogonal to the first two components and so on. The total variance is defined as the sum of the variances of the predicted values of each component (the variance is the square of the standard deviation), and for each component, the proportion of the total variance is computed.

### Creatine determination

CRC cells were seeded into 6-well plates in appropriate growth medium. Upon subconfluence, cells were collected, homogenized and proteins removed using 10 kDa MWCO spin filters. Relative creatine content was assayed using Creatine Assay Kit (Sigma) according to the manufacturer’s instructions and fluorescence intensity measured with a GloMax®-Multi Detection System (Promega, Madison, WI, USA). Values were normalized to total protein content.

### Reduced glutathione measurement

Cells were seeded at a density of 20000 cells per well (30000 in the case of SW620 cells) in a 96-well plate. At 24 hours, medium was removed, cells lysed, and the relative levels of reduced glutathione (GSH) were determined using GSH-GSSG-Glo^TM^ Glutathione Assay (Promega). Luminescence intensity of the samples was measured with a GloMax®-Multi Detection System (Promega).

### Data availability

All data generated or analysed during this study are included in this published article (and its Supplementary Information files).

## Electronic supplementary material

Supplementary Figures

Dataset 1
