# Supplementary material for: Complementary ACSL isoforms contribute to a non-Warburg advantageous energetic status characterizing invasive colon cancer cells
Source: Sci Rep. 2017 Sep 11;7:11143. doi: 10.1038/s41598-017-11612-3 (PMC5593891; doi:10.1038/s41598-017-11612-3)
Supplement: Supplementary file 1 — Supplementary Figures [file 41598_2017_11612_MOESM1_ESM.pdf]

Supplementary Figures

Complementary ACSL isoforms contribute to a non-Warburg advantageous energetic status characterizing invasive colon cancer cells

Ruth Sánchez-Martínez[1*], Silvia Cruz-Gil[1], María Soledad García-Álvarez[1], Guillermo Reglero[1], Ana Ramírez de Molina[1*]

[1]Molecular Oncology and Nutritional Genomics of Cancer Group, IMDEA Food Institute, CEI UAM + CSIC, E28049, Madrid, Spain. *Corresponding Author

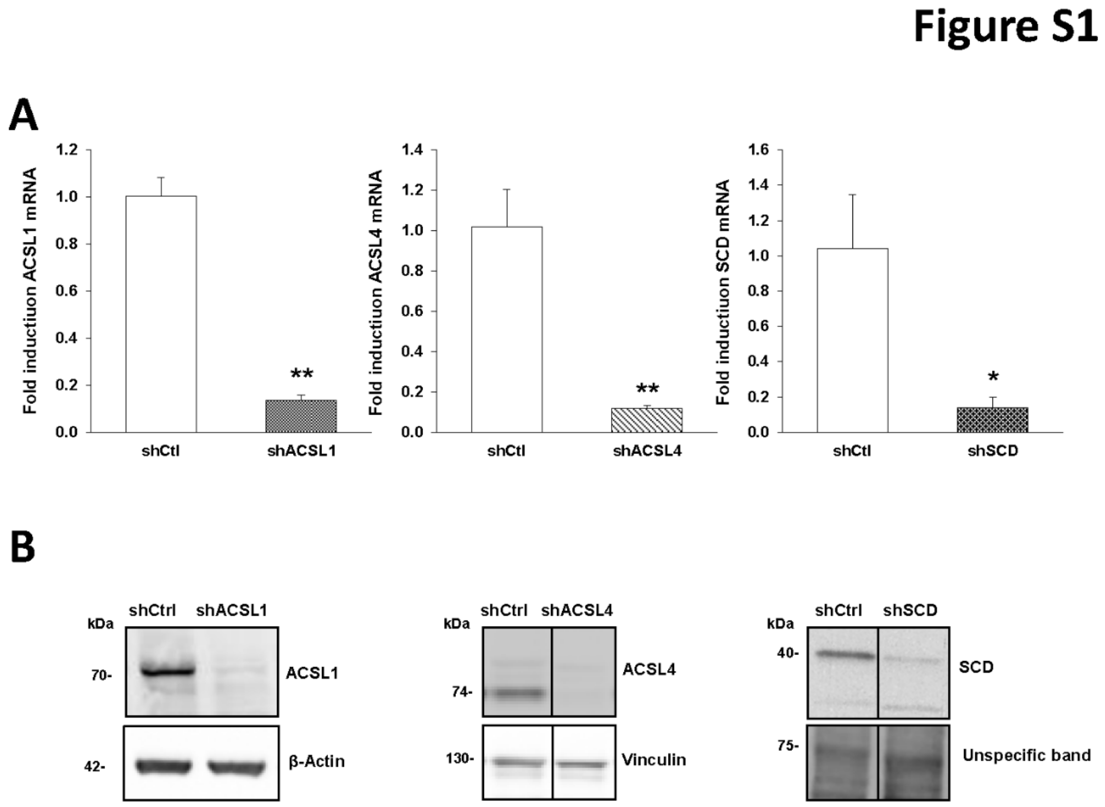

Figure S1. ACSL1, ACSL4 and SCD knock-down by shRNA means. Related to Figure 1,2 and 6.

A. Cell lines stably expressing shRNAs for ACSL1 (shACSL1), ACSL4 (shACSL4), SCD (shSCD) or scramble (shCtrl) were generated using lentiviral transduction and knock-down of every gene was measured by RT-QPCR. B. Protein expression levels of ACSL1 (upper band), ACSL4 and SCD for each cell line were detected by Western Blot with specific antibodies. Vinculin or an unspecific band detection were used as a loading control. Experiments in B were performed in triplicates (n = 3). Results represent the mean ±SD (n = 3). *, $p < 0.05$, **, $p < 0.01$, ***, $p < 0.001$.

Figure S2

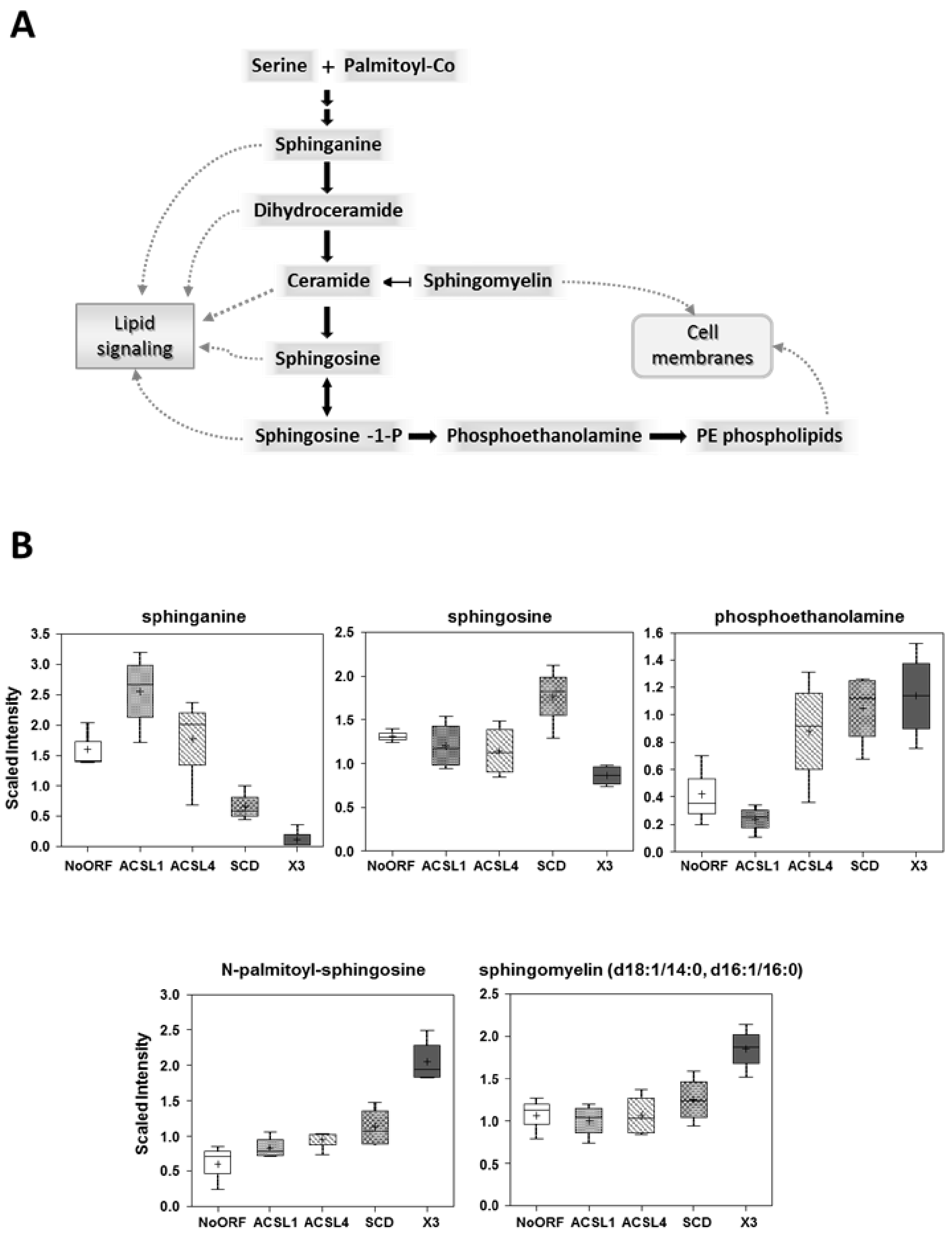

Figure S2. Sphinganine levels are strongly reduced in x3 cells. Related to Figure 5.

A. Schematic representation of sphinganine metabolism. B. Sphinganine and sphingosine levels are reduced in DLD-1 cells expressing ACSL1, ACSL4 and SCD. Accordingly, derived complex lipids such as phosphoethanolamine, the ceramide N-palmitoyl-sphingosine and sphingomyelin are augmented. Box plots show the scaled intensity (Y axis) for each metabolite. Mean, median and maximum and minimum values of the distribution are represented.

Figure S3

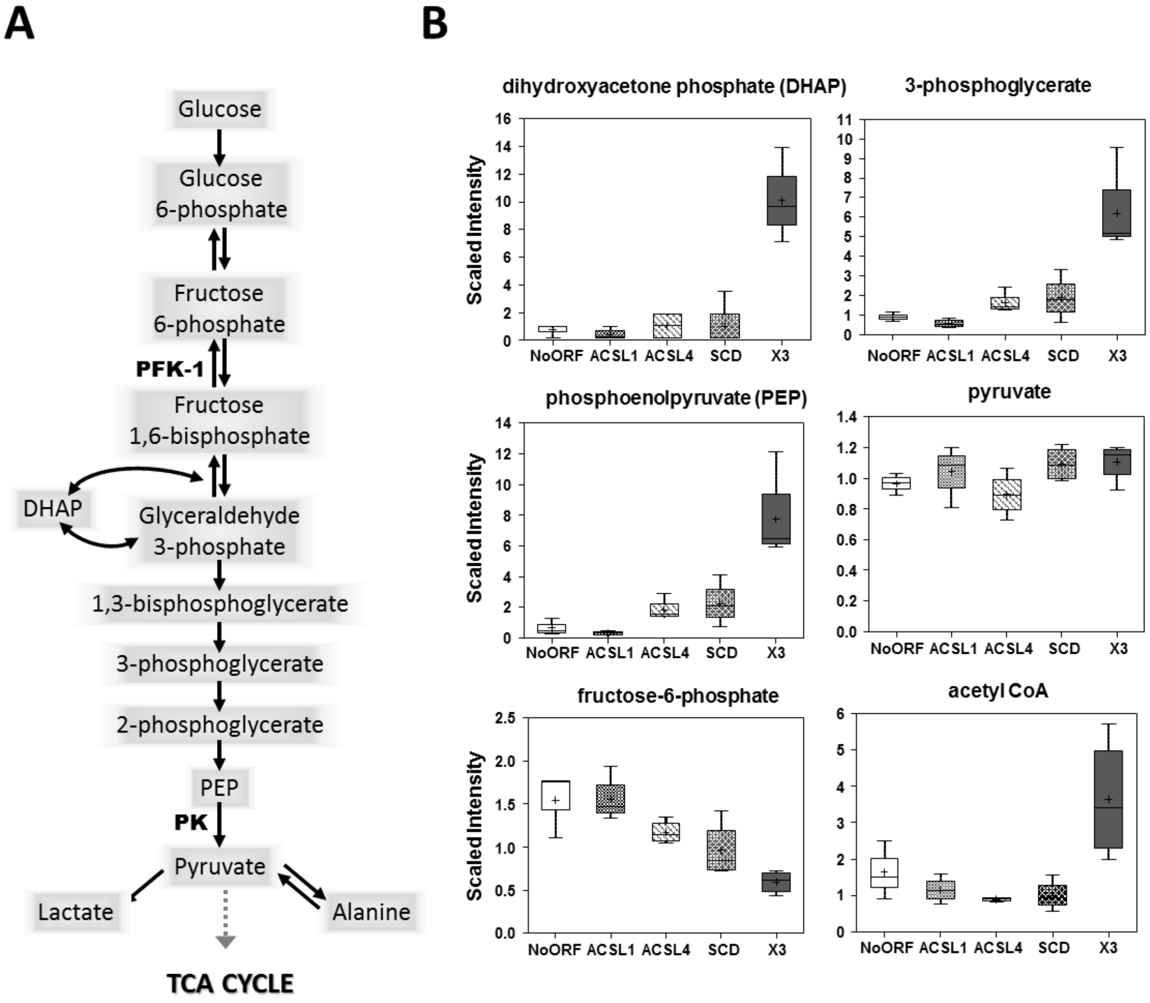

Figure S3. Perturbations in glycolysis pathway in X3 cells. Related to Figure 4 and 5.

A. Schematic representation of glycolysis (DHAP: dihydroxyacetone phosphate; PEP: phosphoenolpyruvate; PFK-1: phosphofructokinase 1; PK: pyruvate kinase). B. Box plots show the scaled intensity (Y axis) for several glycolytic metabolites in the different DLD-1 expression-subtypes. Mean, median and maximum and minimum values of the distribution are represented in the plots.

Figure S4

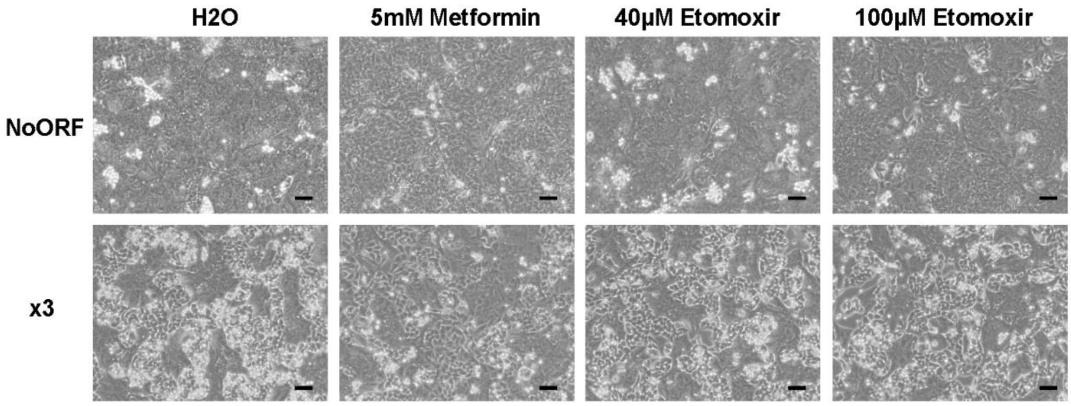

Figure S4. Etomoxir treatment is not able to reverse x3 cells EMT phenotype while metformin treatment rescues normal DLD-1 epithelial phenotype. Related to Discussion.

Representative phase contrast pictures of No ORF and x3 cells treated either with 5 mM metformin, 40mM Etomoxir, 100mM Etomoxir or vehicle (water) for 48 hours. Scale bars, 100 µm.
